# Integrating selective signatures and Cis-eQTLs to prioritize candidate genes potentially related to milk production traits in dual-purpose cattle

**DOI:** 10.3389/fvets.2026.1888422

**Published:** 2026-07-21

**Authors:** Kailun Ma, Xue Li, Menghua Zhang, Dan Wang, Shengchao Ma, Xixia Huang, Qiuming Chen, Lei Xu

**Affiliations:** College of Animal Science, Xinjiang Agricultural University, Urumqi, China

**Keywords:** Chinese Simmental cattle, eQTL, transcriptome sequencing, whole-genome resequencing, Xinjiang brown cattle

## Abstract

This study aimed to identify candidate genes and genetic markers affecting milk production traits in dual-purpose cattle, providing a foundation of data for marker-assisted breeding. Cis-eQTL analysis was performed on 83 Xinjiang brown cattle and 80 Chinese Simmental cattle using whole-genome resequencing and RNA-seq data derived from blood samples. Candidate genes were prioritized by overlapping the eGenes with genes from previous selective signature analyses. In Xinjiang brown cattle, 293,758 cis-eQTLs regulating 1,720 genes were identified, whereas 78,735 cis-eQTLs regulating 569 genes were found in Chinese Simmental cattle. By overlapping the eGenes with candidate genes from previous selective signature analyses, we prioritized 103 genes (e.g., *PPP2R3A, GNAI1*, and *ITGB4*) in Xinjiang brown cattle and 26 candidate genes (e.g., *MAPK10* and *HPSE*) in Chinese Simmental cattle as potentially related to milk production traits. These results provide valuable data for the development of new dairy lines in Xinjiang brown cattle and for future genomic selection in dairy-oriented Chinese Simmental cattle.

## 1 Introduction

Xinjiang is a major livestock production base in China and boasts vast amounts of natural grasslands. The primary dual-purpose cattle breeds in Xinjiang are the Xinjiang brown cattle (XJBC) and Chinese Simmental cattle (CSC), which are independently developed breeds in China that demonstrate excellent performance in both milk and meat production. The development of Xinjiang brown cattle involved the use of local Kazakh cattle as the maternal line, with the introduction of Brown Swiss, Alataw cattle and a small proportion of Kostroma cattle as paternal sources. This process is followed by crossbreeding improvement and long-term selective breeding (1). In contrast, Simmental cattle originated in the Swiss Alps and are renowned as a dual-purpose breed characterized by fast growth, high milk yield, and good meat quality. China began importing Simmental cattle in the early 20th century, and the 1950s marked the onset of large-scale introductions of dairy and beef types from other countries. Notably, some of these animals were purebred and expanded at the Hutubi Cattle Farm in Xinjiang (2).

Gene expression serves as a bridge linking genetic variation to phenotypic outcomes and is itself regulated by genomic variants. As an intermediate phenotype connecting genotype and target traits, the heritability of gene expression enables its analysis via quantitative trait locus (QTL) mapping, similar to conventional trait phenotypes, thereby leading to the identification of expression quantitative trait loci (eQTL) (3, 4). It allows the identification of genetic loci influencing expression abundance, reveals the regulatory mechanisms underlying genetic variation, and has been established as an important approach for understanding the genetics of gene expression (5). On the basis of the physical distance between the genetic variant and the regulated gene, eQTLs are categorized into cis-acting eQTLs (cis-eQTLs) and trans-acting eQTLs (trans-eQTLs) (6). Studies have shown that compared with trans-eQTLs, cis-eQTLs typically exhibit greater genetic effects, and identifying cis-eQTLs provides a more straightforward path to understanding the biological functions of candidate genes (7). For instance, Zheng et al. (8) performed an eQTL analysis using whole-genome sequencing (WGS) data and longissimus dorsi muscle expression profiles from 19 F2-generation hybrid Xiang pigs. They identified a locus, rs319855910, located in the 5′ UTR of the *MIPEP* gene. This locus was found among the genes annotated from cis-acting eSNPs and is a candidate variant influencing pork quality traits. In another study, 828 cis-eQTLs associated with 1,062 genes were revealed in the muscle tissue of Nellore cattle. Genes such as *IFNT3, IFN-TAU*, and *PRKCG* were associated with biological processes related to innate and adaptive immune responses. These genes were also linked to somatic cell scores in cattle (9). Wang et al. identified 1,780 significant SNPs and 1,538 cis-regulated genes. Among these genes, 153 overlapping genes potentially play important roles in cattle body weight traits (10). These regulatory factors can affect phenotypic outcomes by modulating the expression of key genes.

Blood serves as the central medium for substance transport in animals, through which various hormones, nutrients, and signaling molecules reach their target organs to coordinate growth, development, and physiological functions (11). During lactation, prolactin is secreted by the pituitary gland and acts on mammary tissue via blood circulation, promoting the synthesis of milk fat, milk protein, and lactose; nutrients, inorganic salts, and antibodies required for milk synthesis are also transported to the mammary gland through blood (12, 13). Moreover, blood immune cells play critical roles in the onset, progression, and regression of bovine mastitis, with large numbers of immune cells being recruited to mammary tissue and transferred into milk during infection (14). Compared with mammary tissue, blood samples are more easily accessible and less invasive to animals (15), and the gene expression profiles derived from blood provide a new avenue for elucidating the molecular regulatory mechanisms underlying lactation performance. Previous studies have successfully screened candidate genes related to mastitis resistance and somatic cell score using blood transcriptomes (16–18), and small-molecule metabolites in blood have also been shown to serve as biomarkers for high somatic cell count (19). Therefore, eQTL analysis using blood tissue can capture systemic regulatory signals relevant to lactation, providing a foundation for subsequent targeted validation. To date, no eQTL analysis has been reported in populations of Xinjiang brown cattle and Chinese Simmental cattle. Thus, this study aims to identify cis-eQTLs in the blood tissue of these two breeds using genotypic SNP and gene expression data. We will subsequently integrate these results with previously obtained genomic selective signatures from our research group to further screen for candidate genes associated with milk production traits. This work aims to provide a theoretical foundation for the genetic improvement of Xinjiang brown cattle and Chinese Simmental cattle.

## 2 Materials and methods

### 2.1 Experimental materials

A total of 83 Xinjiang brown cattle from the Yili Xin Brown Breeding Farm and 80 Chinese Simmental cattle from Yili Chuangjin Ben Niu Animal Husbandry Co., Ltd. were used as the experimental subjects. All the animals were healthy lactating cows raised under identical feeding and management conditions, with similar lactation stages and parities. A 10 mL blood sample was collected from the tail vein of each individual and stored at −20 °C.

### 2.2 Whole-genome resequencing analysis

#### 2.2.1 Data DNA extraction, library construction, and sequencing

DNA was extracted from blood samples using the phenol-chloroform method. The purity, integrity, and concentration were assessed by agarose gel electrophoresis and a Nanodrop 2000 spectrophotometer, with exact quantification using a Qubit 2.0 fluorometer. Qualified DNA samples were stored at −80 °C, and those that passed quality control were shipped on dry ice to BGI-Shenzhen for whole-genome resequencing. Sequencing was performed on the DNBSEQ-T7 platform (MGI Tech, Shenzhen, China) using paired-end 150-bp reads.

#### 2.2.2 Genome sequencing, data filtering and alignment

The raw sequencing data were first subjected to quality assessment using FastQC (https://www.bioinformatics.babraham.ac.uk/projects/fastqc/). To ensure data quality for downstream analysis, the raw data were subjected to quality control filtering. The paired-end sequencing data (in FASTQ format) were processed using fastp v0.23.4 software (20) to remove adapter sequences and low-quality reads, resulting in high-quality clean reads for subsequent analysis. The filtering parameters were as follows: fastp -i -I -o -O -w 4 -q 20 -n 2 -u 30. The high-quality clean reads were then aligned to the bovine reference genome (ARS-UCD 1.2) using the BWA-MEM algorithm in BWA v0.7.17 (21) with default parameters and the -M option to mark split hits as secondary. The resulting alignment files were subsequently sorted, and PCR duplicates were removed using the SortSam and MarkDuplicates modules in Picard v2.25.5, yielding final high-quality alignment results.

#### 2.2.3 SNP extraction and filtering

Variant calling and filtering were performed using GATK v4.4.0 (22) with the following quality control criteria: QD < 2.0, FS > 60.0, SOR > 3.0, MQ < 40.0, MQRankSum < −12.5, QUAL < 30.0, and ReadPosRankSum < −8.0. Individual chromosome VCF files were merged into a genome-wide VCF file using GATK's MergeVcfs utility. For each breed separately, SNP sites were filtered using the following exclusion criteria: (1) minor allele frequency (MAF) < 0.05; (2) missing rate > 0.20; (3) Hardy-Weinberg equilibrium test *P* value ≤ 1 × 10^−6^; (4) quality score (QUAL) < 30; (5) genotype quality (GQ) < 10; and (6) fewer than two alleles. After removing these sites, the resulting VCF file was converted to PLINK format using VCFtools v0.1.17 (23), followed by further removal of sites with a missing data rate >5% and individuals with a missing data rate >10%. Finally, the high-quality SNP set was annotated against the reference genome using ANNOVAR v2016-02-01 software (24).

### 2.3 Transcriptome analysis

#### 2.3.1 Sequencing data filtering and preprocessing

The raw RNA-seq data were obtained from previous experiments by the research group. BGI-Shenzhen performed library construction and paired-end 150 bp sequencing on the DNBSEQ platform. Adapter sequences and low-quality reads were removed to yield valid data. The raw reads were filtered using SOAPnuke v1.5.6 (25), and reads with >0.1% unknown bases (N), an average quality score < 0.4, >25% adapter contamination, >50 consecutive identical bases (poly-X), or those that were too short or generally low quality were removed. This yielded high-quality clean reads for further analysis.

#### 2.3.2 Sequencing data alignment to the reference genome

The filtered clean reads were first aligned to the bovine reference genome (ARS-UCD1.2) using STAR v2.7.10b (26) with parameters –runThreadN 8, –readFilesCommand zcat, –outFilterMultimapNmax 1 (to retain only uniquely mapped reads), and –outSAMtype BAM Unsorted, with default settings for other parameters. Unmapped reads were then realigned using HISAT2 v2.2.1 (27) with parameters –dta-cufflinks, –no-mixed, –no-discordant, -p 10, and –known-splicesite-infile. The resulting alignment files from both aligners were merged using Picard v3.0.0. Alignment statistics were calculated using SAMtools v1.17 (28). To ensure data quality, samples with an overall alignment rate < 0.6 or fewer than 50 million aligned bases were excluded from subsequent analysis. Using the reference genome's GFF annotation file, transcript assembly and gene quantification were performed with StringTie v2.2.1 (29) using parameters -e -B -p 8 -G, and gene expression levels (TPM) were calculated using a Perl script.

### 2.4 Covariate analysis for eQTL mapping

To assess the impact of genetic variation on gene expression and detect associations between genotype and gene expression, the covariate analysis pipeline from CattleGTEx (30) was adopted. First, gene expression levels in both populations were subjected to quantile normalization. Only genes with an average TPM > 0.1 across all samples (31) were retained, enabling cross-individual comparisons of expression levels and eliminating systematic differences. The data were reformatted and organized; corresponding gene coordinates, gene IDs, and names were extracted from the genome annotation file (ARS-UCD1.2_Btau5.0.1Y.gff) to construct a normalized expression matrix for subsequent eQTL mapping. To account for potential hidden confounders in the expression data and improve the accuracy of the eQTL analysis, the Bayesian-based PEER v1.0 model (32) was used. In this study, 15 PEER factors were selected to achieve interpretability in the gene expression analysis, and these factors were included as covariates. To control for the influence of population structure on eQTL detection, principal components (PCs) derived from the population genotype data were also included as covariates in the analysis model. Following the recommendation of CattleGTEx and considering the sample sizes of the two populations in this study, the top 3 principal components were used in the analysis.

### 2.5 Cis-eQTL analysis

Cis-eQTL mapping was performed using QTLtools v1.2 (33) with the –cis mode and a cis-window of 1 Mb (–window 1000000). Nominal association testing was performed with the –nominal 0.01 parameter, retaining results with a nominal *P* value < 0.01. The –chunk parameter was used to partition the task for parallel processing, thereby improving computational efficiency. A significance threshold of *P* < 1 × 10^−6^ was set to identify significant cis-eQTLs and screen for high-confidence associations. We also performed permutation tests (1,000 permutations) using QTLtools to obtain gene-level empirical *P*-values; however, due to the limited statistical power associated with the moderate sample sizes (83 for XJBC and 80 for CSC), we adopted the nominal threshold of *P* < 1 × 10^−6^ for cis-eQTL discovery. The genomic coordinates (eVariants) of significant cis-eQTL SNPs were annotated using ANNOVAR v2016-02-01 with parameters -protocol refGene -operation g -nastring . -vcfinput. The association between the genomic and transcriptomic data was calculated on the basis of a modified linear regression model (34), as shown below in Equation (1):


$$\begin{array}{l} \text{y}=\beta_{0}+\beta_{1}{\text{X}}_{1}+\beta_{2}{\text{X}}_{2}+\epsilon \end{array}$$ (1)

where *y* is the vector of standardized molecular phenotypes, β_0_ is the intercept, β_1_ is the effect vector of the SNP marker, *X*_1_ is the genotype, β_2_ is the effect vector of the covariates, *X*_2_ is the matrix of covariates, and ϵ is the vector of random residuals.

### 2.6 Integration of selective signatures and Cis-eQTLs

To integrate selective signatures with cis-eQTL results, selection-signature genes were first identified using a sliding-window approach (window size = 50 kb, step = 20 kb) based on *F*_st_ and θπ-ratio. Windows with Z(*F*_st_) in the top 5% and windows with log_2_(θπ-ratio) in the top 5% or bottom 5% were intersected to define putatively selected regions. Genes located within these regions were extracted using the ARS-UCD1.2 genome annotation and designated as selection-signature genes. These genes were then overlapped with the eGenes obtained from cis-eQTL analysis (*P* < 1 × 10^−6^, Section 2.5) by direct gene ID matching. For each overlapping gene, the number of supporting cis-eQTLs was recorded for candidate prioritization.

### 2.7 Candidate gene enrichment analysis

Gene Ontology (GO) and Kyoto Encyclopedia of Genes and Genomes (KEGG) pathway enrichment analyses for the cis-eQTL candidate genes in both cattle breeds were performed using the DAVID online database (https://davidbioinformatics.nih.gov/, accessed on 15 May 2025). The input gene list for enrichment analysis comprised the significant eGenes identified in each breed (1,720 genes for Xinjiang brown cattle and 569 genes for Chinese Simmental cattle). The background gene set consisted of all genes that passed expression filtering in each breed (21,457 genes for Xinjiang brown cattle and 21,613 genes for Chinese Simmental cattle). The Benjamini-Hochberg method was applied for multiple-testing correction, and terms with *P* < 0.05 were considered significantly enriched, with FDR-adjusted values reported in the results. Visualization of the results was conducted using the Bioinformatics.com.cn online platform (https://www.bioinformatics.com.cn/, accessed on 21 May 2025).

## 3 Results

### 3.1 Variant detection based on WGS

#### 3.1.1 Genome data statistics

As shown in Table 1, the average alignment rate was 99.86%, with a mean sequencing depth of 30.22 × for the Xinjiang brown cattle population and 99.75% with 40.49 × for the Chinese Simmental cattle population (Supplementary Table 1).

**Table 1:** Summary of whole-genome sequencing data from Xinjiang brown cattle and Chinese Simmental cattle.

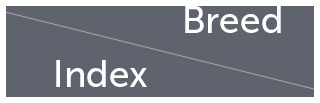	Total reads	Mapped reads	Alignment rate	Total bases	GC content	Mean depth
XJBC	5.61	5.60	99.86%	833.54	42.66%	30.22 ×
CSC	7.56	7.54	99.75%	1,116.68	42.92%	40.49 ×

#### 3.1.2 SNP variant detection and annotation

Following variant calling with GATK, a total of 10,669,376 and 11,722,337 high-quality SNPs were retained for the Xinjiang brown cattle and Chinese Simmental cattle populations, respectively, for subsequent eQTL analysis. The annotation results for these high-quality SNPs in both cattle breeds are summarized in Table 2. Notably, the table shows that the majority of the SNPs were located in intergenic regions, followed by intronic and exonic regions. Furthermore, within exonic regions, most SNPs were classified as either nonsynonymous or synonymous mutations. Specifically, nonsynonymous SNPs alter the amino acid sequence of proteins, whereas synonymous SNPs do not result in a change in the protein sequence.

**Table 2:** Statistics of SNP data for Xinjiang brown cattle and Chinese Simmental cattle.

No.	Region	Count	
		XJBC	CSC
1	Upstream	61,614	70,487
2	Downstream	69,357	76,631
3	Intronic	3870,384	4235,503
4	Intergenic	6488,949	7135,370
5	UTR3	70,468	79,017
6	UTR5	23,240	27,368
7	Splicing	301	349
8	ncRNA	1,224	1,388
9	Exonic	79,249	90,944
10	Other	4,590	5,280
11	Nonsynonymous	25,797	31,112
12	Synonymous	50,169	56,088
13	Stopgain	339	393
14	Stoploss	45	54
15	Unknown	2,931	3,332

The downstream region of the gene is Downstream; the exon region is Exonic; the intergenic region is Intergenic; the intron region is Intronic; the upstream region of the gene is Upstream; the 3′ untranslated region is UTR3; the 5′ untranslated region is UTR5; the splicing site is Splicing; the noncoding RNA is ncRNA; nonsynonymous mutation is Nonsynonymous; synonymous mutation is Synonymous; the codon that encodes the amino acid that is mutated to the stop codon is Stopgain; the stop codon mutated to the codon encoding other types of amino acids is Stoploss; unknown is Unknown. The same applies below.

### 3.2 Transcriptome analysis

#### 3.2.1 Transcriptome sequencing data quality

RNA extracted from the buffy coats of both populations was subjected to transcriptome sequencing on the BGI DNBseq platform. Libraries were constructed using the DNBSEQ eukaryotic mRNA library protocol with a PE150 sequencing strategy. The RNA-seq data underwent correction, quality control, and descriptive statistical analysis. As shown in Table 3, the average number of clean reads was 39,755,617.93 for the Xinjiang brown cattle population (maximum: 40,150,911; minimum: 32,026,254) and 39,929,625.53 for the Chinese Simmental cattle population (maximum: 40,157,656; minimum: 36,514,173). The Q20 and Q30 values were above 97% and 93%, respectively, for both breeds. The GC content was approximately 50%, with a maximum deviation of no more than 6%. These results indicate that the transcriptome sequencing data were of high quality and suitable for subsequent analysis (Supplementary Table 2).

**Table 3:** Transcriptome sequencing data quality of Xinjiang brown cattle and Chinese Simmental cattle.

Breed	Index	Mean	Minimum	Maximum	Standard deviation
XJBC	Clean reads	39755617.93	32026254	40150911	1312867.28
	Clean bases	11900000000	9607876200	12045273300	393900000
	Q20 (%)	97.79	96.53	98.18	0.21
	Q30 (%)	93.13	89.7	94.48	0.66
	GC content (%)	50.32	48.92	51.82	0.80
CSC	Clean reads	39929625.53	36514173	40157656	667950.22
	Clean bases	12000000000	10954251900	12047296800	200400000
	Q20 (%)	98.56	98.12	98.86	0.11
	Q30 (%)	93.96	92.3	95.12	0.42
	GC content (%)	47.77	45.95	50.66	1.14

#### 3.2.2 Transcriptome reference genome alignment analysis

The alignment statistics of the transcriptome sequencing data against the reference genome for the two breeds are shown in Table 4. The average alignment rates were 95.46% for the Xinjiang brown cattle population and 96.25% for the Chinese Simmental cattle population, indicating high sequencing quality and reliability and confirming the suitability of the data for subsequent analysis (Supplementary Table 3).

**Table 4:** Transcriptome sequencing data reference genome alignment of Xinjiang brown cattle and Chinese Simmental cattle.

Breed	Index	Mean	Minimum	Maximum	Standard deviation
XJBC	Uniq mapped	37949106.14	30637049	38541033	1261822.69
	Uniq map rate (%)	95.46	93.19	96.30	0.50
	Mult mapped	926637.49	677821	1969078	173594.88
	Mult mapped rate (%)	2.33	1.88	4.91	0.42
CSC	Uniq mapped	38430934.56	35122961	38950756	688909.65
	Uniq map rate (%)	96.25	94.02	97.13	0.69
	Mult mapped	894498.8	663569	1507080	175440.59
	Mult mapped rate (%)	2.24	1.65	3.76	0.43

#### 3.2.3 Expression level normalization

On the basis of the RNA-seq data from Xinjiang brown cattle and Chinese Simmental cattle, TPM files from all individuals within each breed were merged using a Perl script to generate initial gene expression matrices. Low-expression genes (average TPM ≤ 0.1 across all individuals) were filtered out. The filtered gene expression matrices were then subjected to quantile normalization using the quantile_normalization function in R. The transcript types of the normalized genes were annotated on the basis of the reference genome's GFF file. A total of 21,457 genes were retained in Xinjiang brown cattle, of which 15,896 (74.08%) were protein-coding genes and 3,157 (14.71%) were long noncoding RNAs (lncRNAs). With respect to Chinese Simmental cattle, 21,613 genes were retained, comprising 15,935 (73.73%) protein-coding genes and 3,240 (14.99%) long noncoding RNAs (lncRNAs) (Table 5). Consequently, 21,457 and 21,613 genes from Xinjiang brown cattle and Chinese Simmental cattle, respectively, were used for the subsequent eQTL analysis.

**Table 5:** Transcript types of genes after filtration.

Gene type	XJBC	CSC
lncRNA	3,157 (14.71%)	3,240 (14.99%)
miRNA	440 (2.05%)	445 (2.06%)
Protein-coding	15,896 (74.08%)	15,935 (73.73%)
snoRNA	422 (1.97%)	435 (2.01%)
snRNA	482 (2.25%)	484 (2.24%)
tRNA	751 (3.50%)	765 (3.54%)
Other	309 (1.44%)	309 (1.43%)
Total	21,457	21,613

### 3.3 Cis-eQTL analysis based on high-throughput sequencing

#### 3.3.1 Cis-eQTL detection

**Figure 1:**
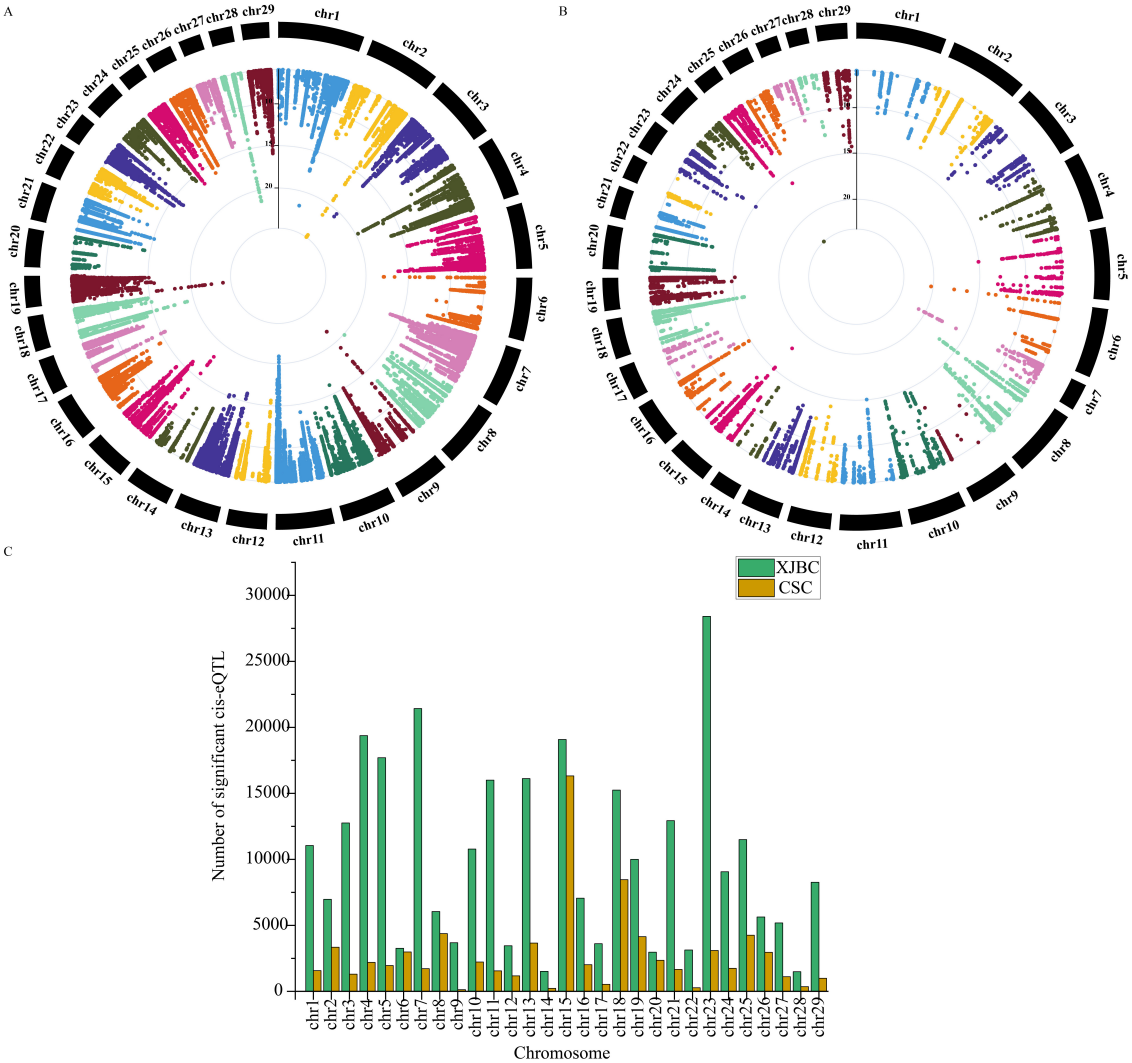
Cis-eQTLs of Xinjiang brown cattle and Chinese Simmental cattle. **(A)** CMplot of cis-eQTLs for Xinjiang brown cattle. **(B)** CMplot of cis-eQTLs for Chinese Simmental cattle. **(C)** Number of significant cis-eQTLs on different chromosomes in Xinjiang brown cattle and Chinese Simmental cattle.

As shown in Table 6, a total of 293,758 significant cis-eQTLs (*P* < 1 × 10^−6^), annotated to 1,720 genes, were identified in the Xinjiang brown cattle population (Supplementary Table 4). In the Chinese Simmental cattle population, 78,735 significant cis-eQTLs, annotated to 569 genes, were identified (Supplementary Table 5). All these eQTLs were located on autosomes. The number of significant cis-eQTLs was much greater than the number of genes, suggesting either that many SNPs are in strong linkage disequilibrium or that multiple variants exist within a single gene. Manhattan plots illustrating the genomic distribution of significant cis-eQTLs are presented in Figures 1A, B. The most significant cis-eQTL in Xinjiang brown cattle was located on chromosome 2 (rs443435653, *P* = 1.92 × 10^−25^), which is associated with the candidate gene *DNER*. In Chinese Simmental cattle, the most significant cis-eQTL was located on chromosome 24 (rs382929226, *P* = 6.05 × 10^−24^), associated with the candidate gene *STARD6*. The distribution of significant cis-eQTLs across different chromosomes for both breeds is shown in Figure 1C. In Xinjiang brown cattle, chromosome 23 contained the greatest number of cis-eQTLs (28,407), involving 118 genes, while chromosome 28 contained the fewest (1,490), involving 15 genes. In Chinese Simmental cattle, chromosome 15 contained the greatest number of cis-eQTLs (16,321), involving 36 genes, whereas chromosome 9 contained the fewest (132), involving 10 genes.

**Table 6:** Summary information of significant cis-eQTLs in two breeds of cattle (*P* < 1 × 10^−6^).

Breed	eQTL number	Gene number	eQTL number/gene number
XJBC	293,758	1,720	170.79
CSC	78,735	569	138.37

#### 3.3.2 Cis-eQTL functional annotation

Functional annotation of the SNPs identified from the top cis-eQTLs in both breeds was performed using ANNOVAR software. The results are shown in Table 7. With respect to Xinjiang brown cattle, the majority of eQTL SNPs were located in intronic regions (47.797%), followed by intergenic regions (42.571%). Exonic regions accounted for 2.471% of the SNPs, comprising 2,580 nonsynonymous mutations and 4,383 synonymous mutations. For Chinese Simmental cattle, most eQTL SNPs were also located in intronic regions (53.554%), followed by intergenic regions (37.512%). Exonic regions accounted for 2.441% of the SNPs, which included 680 nonsynonymous mutations and 1,185 synonymous mutations.

**Table 7:** Functional annotation of significant cis-eQTL loci.

Annotation category	XJBC	CSC
Downstream	5,630 (1.917%)	1,350 (1.715%)
Intergenic	125,057 (42.571%)	29,535 (37.512%)
Intronic	140,408 (47.797%)	42,166 (53.554%)
Upstream	5,468 (1.861%)	1,273 (1.617%)
UTR3	5,822 (1.982%)	1,621 (2.059%)
UTR5	2,543 (0.866%)	695 (0.883%)
Splicing	36 (0.012%)	16 (0.020%)
Others	1,536 (0.523%)	157 (0.199%)
Nonsynonymous	2,580 (0.878%)	680 (0.862%)
Synonymous	4,383 (1.491%)	1,185 (1.503%)
Stopgain	37 (0.013%)	13 (0.016%)
Stoploss	8 (0.003%)	5 (0.006%)
Unknown	256 (0.087%)	42 (0.053%)

#### 3.3.3 Functional enrichment analysis of eGenes

**Figure 2:**
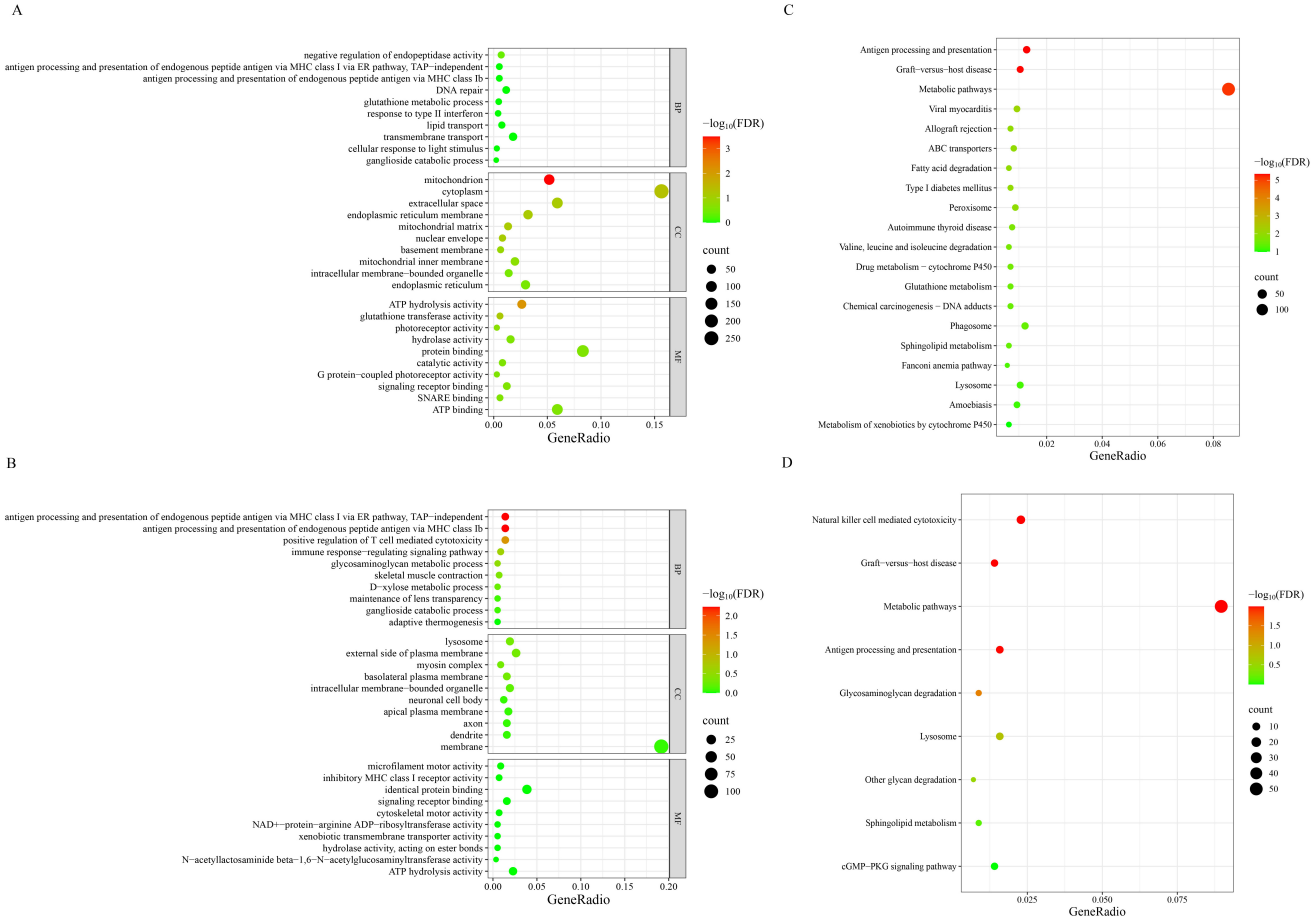
Functional enrichment analysis of cis-eQTL genes. **(A)** Results of the GO enrichment analysis of eGenes in Xinjiang brown cattle. **(B)** Results of the GO enrichment analysis of eGenes in Chinese Simmental cattle. **(C)** KEGG enrichment results of eGenes from Xinjiang brown cattle. **(D)** KEGG enrichment results of eGenes from Chinese Simmental cattle. The x-axis represents GeneRatio, dot size represents gene count (Count), and dot color represents FDR-adjusted *P*-values (Benjamini-Hochberg correction). Only terms with nominal *P* < 0.05 are shown.

Functional enrichment analysis of eGenes identified from cis-eQTLs in both Xinjiang brown cattle and Chinese Simmental cattle was performed using DAVID. As shown in Figures 2A, B, the results of the GO enrichment analysis revealed that eGenes in Xinjiang brown cattle were enriched in 102 terms at the nominal significance level (*P* < 0.05). After Benjamini-Hochberg FDR correction, 3 terms (mitochondrion, ATP hydrolysis activity, and cytoplasm) remained significant (FDR < 0.05). These included 47 terms in the biological process category, such as negative regulation of endopeptidase activity, antigen processing and presentation of endogenous peptide antigen via MHC class I via the ER pathway (TAP-independent), and antigen processing and presentation of endogenous peptide antigen via MHC class Ib. In the cellular component category, 19 terms were enriched, including mitochondrion, cytoplasm, and extracellular space. The molecular function category was enriched in 36 terms, including ATP hydrolysis activity, glutathione transferase activity, and photoreceptor activity. Among all the terms, mitochondria was the most significantly enriched, with 73 genes. For Chinese Simmental cattle, eGenes were enriched in 43 GO terms at the nominal level (*P* < 0.05), of which 3 terms (antigen processing and presentation of endogenous peptide antigen via MHC class I via ER pathway, TAP-independent; antigen processing and presentation of endogenous peptide antigen via MHC class Ib; and positive regulation of T cell mediated cytotoxicity) remained significant after FDR correction (FDR < 0.05). These included 18 terms in the biological process category, such as antigen processing and presentation of endogenous peptide antigen via MHC class I via the ER pathway (TAP-independent), antigen processing and presentation of endogenous peptide antigen via MHC class Ib, and positive regulation of T-cell-mediated cytotoxicity. The cellular component category contained 14 enriched terms, including the lysosome, the external side of the plasma membrane, and the myosin complex. The molecular function category contained 11 enriched terms, including microfilament motor activity, inhibitory MHC class I receptor activity, and identical protein binding. The most significant term across all categories was the antigen processing and presentation of endogenous peptide antigens via MHC class I via the ER pathway (TAP-independent), which was enriched with 8 genes.

The KEGG enrichment analysis results for the eGenes of Xinjiang brown cattle and Chinese Simmental cattle are presented in Figures 2C, D. The eGenes of Xinjiang brown cattle were enriched in 39 pathways at the nominal level (*P* < 0.05), of which 14 pathways (including Antigen processing and presentation, Graft-vs.-host disease, Metabolic pathways, Viral myocarditis, Allograft rejection, ABC transporters, Fatty acid degradation, Type I diabetes mellitus, Peroxisome, Autoimmune thyroid disease, Valine, leucine and isoleucine degradation, Drug metabolism—cytochrome P450, Glutathione metabolism, and Chemical carcinogenesis—DNA adducts) remained significant after FDR correction (FDR < 0.05). The eGenes of Chinese Simmental cattle were enriched in 9 pathways at the nominal level (*P* < 0.05), of which 5 pathways (Natural killer cell mediated cytotoxicity, Graft-vs.-host disease, Metabolic pathways, Antigen processing and presentation, and Glycosaminoglycan degradation) remained significant after FDR correction (FDR < 0.05).

### 3.4 Integration of selective signatures and Cis-eQTLs to mine candidate genes

**Figure 3:**
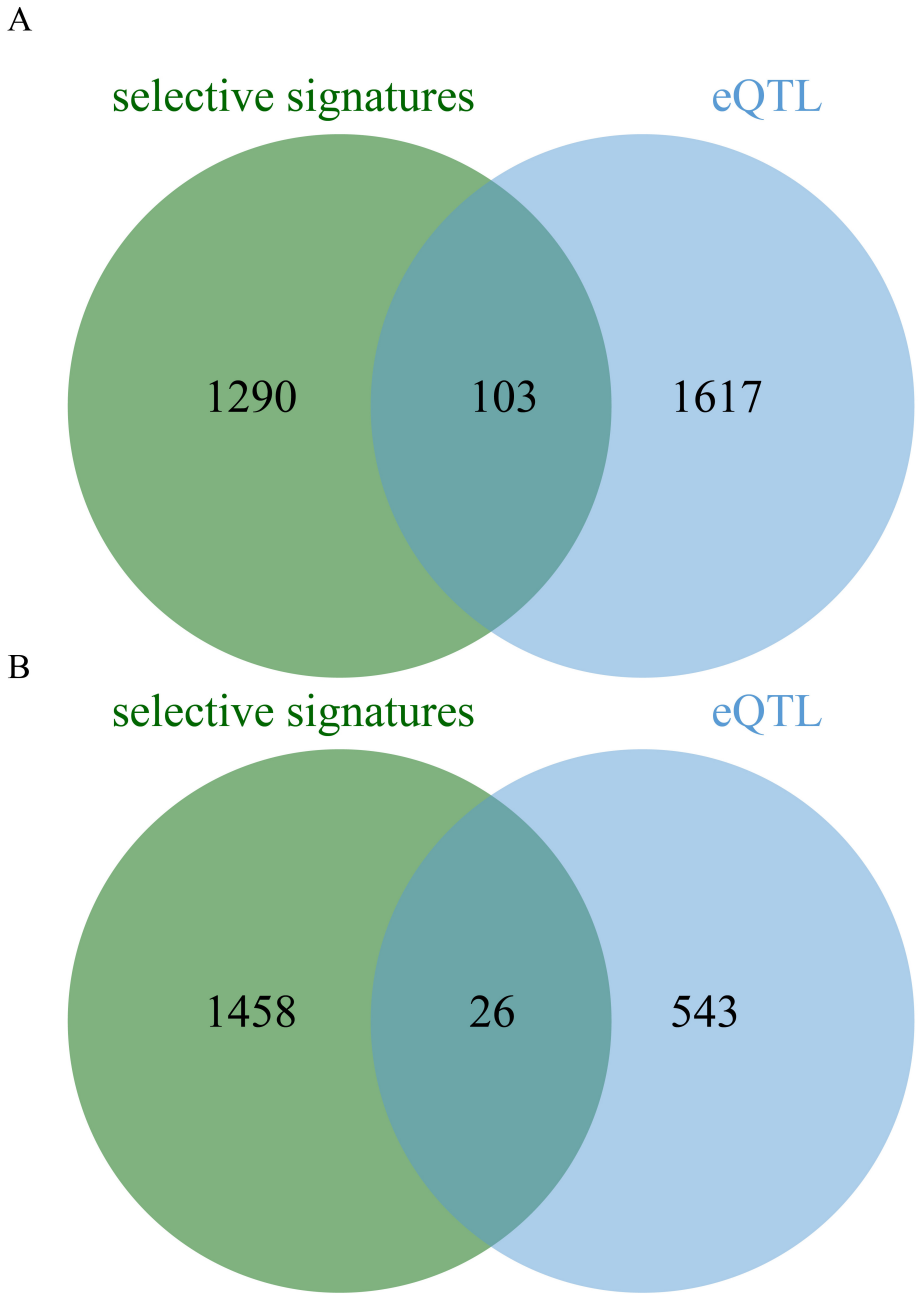
Shared common candidate genes of selective signatures and cis-eQTLs. **(A)** Common candidate genes of Xinjiang brown cattle. **(B)** Common candidate genes of Chinese Simmental cattle.

An overlap analysis was performed between the candidate genes identified from prior selective signature analyses (35) and those derived from significant cis-eQTLs. The results are shown in Figures 3A, B. On the basis of the results of the cis-eQTL analysis, 1,720 genes were identified in Xinjiang brown cattle, and 569 genes were identified in Chinese Simmental cattle. By integrating these results with previous findings on selective signatures, prioritized candidate genes potentially associated with milk production traits were further screened. To assess the statistical significance of this overlap, we performed hypergeometric tests. The results showed that the overlap did not exceed random expectation in either breed (XJBC: *P* = 0.81; CSC: *P* = 0.991), indicating that the overlapping genes should be interpreted as exploratory or prioritized candidates rather than definitive associations. This integrated approach identified 103 milk production trait-related genes (containing 18,959 cis-eQTLs) in Xinjiang brown cattle. Among these genes, genes such as *PPP2R3A, TRNAY-GUA, TRNAC-GCA, GNAI1, TRNAI-AAU*, and *ITGB4* have been previously reported in the literature to be associated with milk production (Supplementary Table 6). For Chinese Simmental cattle, 26 candidate genes associated with milk production traits (containing 3,149 cis-eQTLs) were identified, including *HPSE, MAPK10, TRNAY-GUA*, and *TRNAC-GCA*, which have also been reported to be related to milk production traits (Supplementary Table 7).

## 4 Discussion

In this study, eQTL association analysis was conducted using gene expression data from 83 Xinjiang brown cattle and 80 Chinese Simmental cattle. We identified 293,758 significant cis-eQTLs corresponding to 1,720 genes in Xinjiang brown cattle and 78,735 significant cis-eQTLs corresponding to 569 genes in Chinese Simmental cattle (*P* < 1 × 10^−6^). The number of significant cis-eQTLs far exceeded the number of genes, which is likely attributable to linkage disequilibrium (LD) among nearby SNPs and regional correlation, rather than pleiotropic effects. Notably, the number of cis-eQTLs detected in Xinjiang brown cattle was approximately four times that in Chinese Simmental cattle. This disparity can be largely explained by differences in linkage disequilibrium (LD) between the two breeds. Our independent LD analysis revealed that Xinjiang brown cattle have higher mean LD (r^2^ = 0.1084 at 100 kb) and slower LD decay compared to Chinese Simmental cattle (r^2^ = 0.0993 at 100 kb) (35). Higher LD in Xinjiang brown cattle would cause more tag SNPs to be associated with the same causal variant, inflating the number of significant cis-eQTLs. In contrast, the faster LD decay and higher genetic diversity in Chinese Simmental cattle result in a sparser set of significant associations, suggesting that eQTL signals in this breed are more refined and potentially enriched for causal variants. Therefore, the observed difference in eQTL yield primarily reflects distinct genetic architectures rather than technical artifacts or differences in experimental conditions.

To further investigate whether the substantial difference in cis-eQTL discovery between the two breeds (293,758 in XJBC vs. 78,735 in CSC) reflects genuine differences in genetic architecture, we systematically compared multiple genetic parameters between the two breeds. Genome-wide MAF distributions showed that the mean MAF values were similar (0.239 for XJBC vs. 0.230 for CSC), with broadly similar overall distributions, though CSC had a slightly higher proportion of SNPs in the intermediate MAF range (0.1–0.3), consistent with its higher genetic diversity (35). The average number of variants tested per gene did not differ significantly between breeds, and the numbers of genes retained after expression filtering were similar (21,457 vs. 21,613), but eGenes in XJBC exhibited slightly higher expression variance. The most notable difference was observed in LD decay patterns, which directly explains why XJBC has far more cis-eQTLs than CSC. To further distinguish shared regulatory signals from breed-specific eQTLs, we compared the eGene lists between breeds and found 230 shared eGenes, accounting for 13.4% of XJBC eGenes and 40.4% of CSC eGenes, indicating that most eQTL regulatory signals are breed-specific. Notably, the shared eGenes include multiple genes previously reported to be associated with lactation traits (e.g., *PPP2R3A, GNAI1, ITGB4, MAPK10, HPSE*), suggesting that these cross-breed conserved signals may represent core lactation-related regulatory genes worthy of prioritized validation in future studies. In summary, the breed difference in eQTL discovery is primarily attributable to differences in LD architecture rather than technical artifacts or statistical false positives.

In Xinjiang brown cattle, the significant cis-eQTLs were predominantly distributed on autosomes 23, 7, 4, 15, and 5, with chromosome 23 containing a markedly greater number of cis-eQTLs than the other autosomes had. In Chinese Simmental cattle, the significant cis-eQTLs were located mainly on autosomes 15, 18, 8, 25, and 19, with chromosome 15 harboring a significantly greater number of cis-eQTLs. The distinct chromosomal enrichment patterns between the two breeds likely result from multiple factors. First, as discussed above, differences in LD architecture play a key role: higher LD in Xinjiang brown cattle causes a single causal variant to drag many tagging SNPs into significance, inflating eQTL counts on certain chromosomes, while the lower LD in Chinese Simmental cattle yields sparser but more refined signals. Second, chromosome-specific selection pressures may differ between the two breeds—Xinjiang brown cattle may have been selected for milk-associated loci on chromosome 23, while Chinese Simmental cattle may have experienced similar selection on chromosome 15. Third, breed-specific regulatory variants may contribute, as certain eQTLs may be present only in one breed and cluster on particular chromosomes. Additionally, variations in SNP density, gene density, and local LD structures across chromosomes may also influence statistical power and thus the number of eQTLs detected per chromosome. A growing body of research suggests that SNP loci associated with traits, identified through whole-genome resequencing, often overlap with cis-eQTL regions (36). Conducting expression quantitative trait locus analysis thus provides further support for the prior selection signature results related to the milk production traits identified in this study. Integrating selective sweeps with gene expression data or eQTL association analysis enables more efficient screening of candidate genes that regulate important economic traits and reveals potential underlying mechanisms (37, 38). Friedrich et al. conducted a joint analysis of selection signals and significant cis-eQTLs from 22 different tissues in the Cattle GTEx database using eight cattle breeds from Africa. Their results indicated that the *NDUFS3* gene is associated with heat tolerance in African indigenous cattle breeds, and *GIMAP8* was identified as a candidate gene for tick resistance in cattle (39). Liu et al. performed genomic selection signature and runs of homozygosity (ROH) analyses on Holstein cattle. They reported that among 256 genes within iHS genomic regions, 81 eGenes were significantly expressed in trait-relevant tissues, corresponding to 352 cis-eQTLs (across 21 tissues) and 27 trans-eQTLs (across 6 tissues). Furthermore, 1,092 SNP loci within ROH regions overlapped with 108 cis-eQTLs and 4 trans-eQTLs across 13 tissues (40). This integrated approach, which combines signature detection for selection and eQTL analysis, will significantly facilitate the identification of candidate genes for important economic traits and elucidate their regulatory mechanisms.

With respect to milk production traits, this study identified 103 overlapping genes (e.g., *PPP2R3A, GNAI1*, and *ITGB4*) in Xinjiang brown cattle and 26 overlapping genes (e.g., *MAPK10* and *HPSE*) in Chinese Simmental cattle by integrating genomic selective signatures with cis-eQTL analysis. Notably, *TRNAC-GCA* and *TRNAY-GUA* were identified as candidate genes in both breeds and exhibited significant cis-eQTL effects. In terms of lactation regulation, multiple tRNA clusters (*TRNAC-GCA, TRNAY-GUA*, and *TRNAI-AAU*) have been associated with milk production (41). Laodim et al. (42) using GWAS, provided further support for the association of *PPP2R3A* and *GNAI1* with milk production in a multibreed dairy cattle population in Thailand. Ibeagha-Awemu et al. (43) identified *ITGB4* as a novel potential candidate gene associated with bovine milk production traits or mammary gland function through SNP-based genome-wide analysis using high-throughput sequencing data from 1,246 Canadian Holstein cows. Rekik et al. who conducted a GWAS on 308 crossbred dairy cows from different regions of Ethiopia using high-density SNP chip data, identified genetic markers associated with major milk production traits, including the *HPSE* gene located within a significantly associated SNP region (44). Furthermore, *MAPK10* has been associated with milk production in a multibreed Thai dairy cattle population (42). Research has also indicated that *MAPK10* and *TRNAG-CCC* are directly involved in inflammatory processes triggered by mastitis and are potential candidate genes related to the somatic cell count (45). A study by Lu et al. (46) suggests that *MAPK10* may be involved in the occurrence and regulation of mastitis.

To assess whether the observed overlap between selection-signature genes and eGenes exceeded random expectation, we performed hypergeometric tests. For Xinjiang brown cattle, with 1,393 selection-signature genes, 1,720 eGenes, and a background gene set of 21,457 genes (i.e., all expressed genes included in the eQTL analysis), the observed overlap was 103 genes, with an expected overlap of approximately 111.7 genes, yielding a hypergeometric *P*-value of 0.81, which was not significant. For Chinese Simmental cattle, with 1,484 selection-signature genes, 569 eGenes, and a background gene set of 21,613 genes, the observed overlap was 26 genes, with an expected overlap of approximately 39.1 genes, yielding a *P*-value of 0.991, also not significant. These results indicate that in both breeds, the overlap between eGenes and selection-signature genes did not exceed random expectation. The lack of statistical significance may be attributed to several factors: first, selective signatures reflect long-term, multi-directional selection history rather than specifically indicating dairy selection; second, blood eQTLs capture systemic regulatory signals that may differ from mammary-specific selection signals at the tissue level; and third, the limited sample size may have reduced statistical power. Consistent with these results, the overlapping genes identified in this study should be regarded as exploratory or prioritized candidates for milk production traits rather than as definitively associated genes. Future studies with larger cohorts and functional experiments are needed to validate their roles.

Although these genes have been supported by independent GWAS and QTL studies, their biological functions in the context of lactation remain to be further explored. Here, we provide a brief functional interpretation of these prioritized candidates. The cis-eQTL-associated genes identified in blood in this study, such as *PPP2R3A, GNAI1, ITGB4*, and *MAPK10*, although derived from blood transcriptomes, are supported by existing evidence suggesting potential biological links to mammary gland functional pathways. Specifically, *PPP2R3A* encodes a regulatory subunit of protein phosphatase 2A, an enzyme involved in prolactin signaling and the regulation of mammary epithelial cell proliferation. *GNAI1* encodes an inhibitory G-protein α subunit that participates in GPCR-mediated signal transduction and may indirectly influence mammary metabolism through endocrine signaling. As mentioned above, Laodim et al. (42) confirmed significant associations of *PPP2R3A* and *GNAI1* with milk production traits in a GWAS study. *ITGB4* encodes integrin subunit β4, and previous studies have demonstrated its upregulation by mitogens during bovine mammary development (47), indicating a direct role of this gene in mammary tissue development and functional maintenance. *MAPK10* encodes JNK3, a key component of the MAPK signaling pathway. Multiple studies have associated *MAPK10* with mastitis resistance and somatic cell score in dairy cattle (45, 46), suggesting that it may influence lactation performance through immuno-inflammatory regulation. Collectively, these genes may indirectly contribute to the regulation of lactation traits via hormone signal transduction, mammary development, and immune-metabolic regulation.

The results of this study should be interpreted with several considerations. First, there are significant functional differences between the blood tissue used in this study and mammary tissue. Blood eQTLs reflect systemic regulatory signals rather than the direct regulatory mechanisms of milk synthesis in mammary epithelial cells; therefore, cis-eQTLs identified in blood cannot be directly equated with mammary-specific regulatory effects. The enrichment results of this study showed that blood eQTL-associated genes were predominantly enriched in immune-related pathways (e.g., antigen processing and presentation, MHC class I-related processes, natural killer cell-mediated cytotoxicity, etc.). This finding is not unexpected, as blood tissue itself is primarily composed of immune cells, and its transcriptome naturally reflects the host's immune status. Consequently, the blood eQTLs identified in this study are more directly relevant to immune regulation, inflammatory response, mastitis resistance, and somatic cell score, rather than directly reflecting milk synthesis mechanisms in mammary epithelial cells. Second, although we identified significant cis-eQTLs for genes such as *PPP2R3A, GNAI1, ITGB4*, and *MAPK10*, a single gene was often associated with multiple significant eQTL variants, and due to linkage disequilibrium, the effect directions of these variants were not always consistent, making it difficult to assign a unified effect direction at the gene level. Therefore, we did not report a generalized eQTL effect direction for individual genes. Third, we did not systematically query the expression levels of these genes in mammary tissue using public databases such as CattleGTEx, which represents a limitation of our study. Fourth, *TRNAY-GUA* and *TRNAC-GCA* were identified as candidate genes in both breeds. Given that tRNAs belong to multi-copy gene families and are susceptible to annotation and mapping artifacts, their interpretation requires caution. However, their identification as candidates in both breeds reduces the likelihood of false positives to some extent, though their functions still require independent experimental confirmation. Fifth, we acknowledge that our cis-eQTL discovery was based on a nominal P-value threshold (*P* < 1 × 10^−6^) rather than permutation-based gene-level thresholds followed by FDR correction. This approach, while commonly used in exploratory eQTL studies with moderate sample sizes, may have introduced some false positives or failed to capture weak regulatory effects. Given the limited sample sizes (83 for XJBC and 80 for CSC), permutation-based approaches would have suffered from reduced statistical power and potentially missed genuine associations. Nevertheless, we recognize that future studies with larger cohorts should employ permutation testing and FDR correction to validate and refine our findings.

In summary, leveraging the accessibility of blood tissue and its representation of systemic regulatory information, we performed a genome-wide screening of candidate genes potentially associated with lactation. These candidate genes may indirectly influence lactation performance through the immune-metabolic axis—that is, by modulating the host's immune status and inflammatory responses, thereby affecting udder health and lactation persistence, and consequently influencing milk production traits. The results of this study should be interpreted as a preliminary screening of candidate genes rather than definitive conclusions regarding gene function. The specific functions of these candidate genes in mammary tissue require further validation in independent cohorts and functional experiments.

## 5 Conclusion

In this study, cis-eQTL analysis was performed on blood tissue samples from Xinjiang brown cattle and Chinese Simmental cattle using QTLtools software. A total of 1,720 genes were identified in Xinjiang brown cattle, and 569 genes were identified in Chinese Simmental cattle. By further integrating these results with prior genomic selective signatures, 103 candidate genes associated with milk production traits (e.g., *PPP2R3A, TRNAY-GUA, TRNAC-GCA, GNAI1, TRNAI-AAU*, and *ITGB4*) were screened in Xinjiang brown cattle, and 26 candidate genes (e.g., *HPSE, MAPK10, TRNAY-GUA*, and *TRNAC-GCA*) were identified in Chinese Simmental cattle. This study provides a valuable foundation for further screening of candidate genes related to milk production traits and offers an important theoretical basis for the breeding of milk production traits in Xinjiang brown cattle and Chinese Simmental cattle.

## Data Availability

The datasets presented in this study can be found in online repositories. The whole-genome resequencing data and RNA-seq data generated in this study have been deposited in the NCBI Sequence Read Archive (SRA) database under BioProject accession numbers PRJNA1346832 (whole-genome resequencing) and PRJNA1418328 (transcriptome sequencing).

## Supplementary material

Supplementary Table 1 Statistical results of the genomic BAM files for 83 Xinjiang brown cattle and 80 Chinese Simmental cattle.

Supplementary Table 2 Quality statistics of transcriptome sequencing data for 83 Xinjiang brown cattle and 80 Chinese Simmental cattle.

Supplementary Table 3 Alignment analysis of transcriptome sequencing data for 83 Xinjiang brown cattle and 80 Chinese Simmental cattle with the reference genome.

Supplementary Table 4 Summary statistics of significant cis-eQTLs in Xinjiang brown cattle.

Supplementary Table 5 Summary statistics of significant cis-eQTLs in Chinese Simmental cattle.

Supplementary Table 6 Integrated selective signatures and cis-eQTL shared candidate genes for Xinjiang brown cattle.

Supplementary Table 7 Integrated selective signatures and cis-eQTL shared candidate genes for Chinese Simmental cattle.
